# Bullying Victimization and Life Satisfaction Among Rural Left-Behind Children in China: A Cross-Sectional Study

**DOI:** 10.3389/fped.2021.671543

**Published:** 2021-07-30

**Authors:** Yide Yang, Chanjuan Zheng, Ming Xie, Shuqian Yuan, Yuan Zeng, Meiling Zhou, Shuzhen Huang, Yulian Zhu, Xiangli Ye, Zhiyong Zou, Ying Wang, Julien Steven Baker

**Affiliations:** ^1^Key Laboratory of Molecular Epidemiology of Hunan Province, School of Medicine, Hunan Normal University, Changsha, China; ^2^Hunan Preventive and Treatment Center for Occupational Diseases, Changsha, China; ^3^Institute of Child and Adolescent Health, School of Public Health, Peking University, Beijing, China; ^4^Department of Psychiatry, Renmin Hospital of Wuhan University, Wuhan, China; ^5^Department of Sport, Physical Education and Health, Centre for Health and Exercise Science Research, Hong Kong Baptist University, Hong Kong, China

**Keywords:** bullying victimization, life satisfaction, primary school, interactive effects, left behind children

## Abstract

**Objectives:** This study aimed to evaluate the associations between bullying victimization and life satisfaction in primary school children and also investigate the interactive effects of left-behind status and bullying victimization on life satisfaction.

**Materials and Methods:** Bullying victimization was measured using the Chinese version of the revised Olweus Bully/Victim Questionnaire. Life satisfaction was assessed using the Multidimensional Students' Life Satisfaction Scale (MSLSS). Life satisfaction is composed of five domains, namely, family, school, friends, environment, and self-satisfaction. Left-behind status of rural children was defined as one or both their parents migrating to working in cities. The data were analyzed using Mann–Whitney *U* tests, Chi-square tests, and multivariate linear and logistic regression analyses.

**Results:** A total of 810 primary school children were involved, of which 8.5% reported bullying victimization, and 44.3% were left-behind children (LBC). We found that bullying victimization was negatively associated with all domains of life satisfaction (all *p* < 0.05). With further left-behind status-stratified analysis, we found that negative association between bullying victimization and friend satisfaction was more profound in the LBC group than in the non-LBC group [b(SE)= −0.133 (0.03) vs. −0.061 (0.026) for LBC and non-LBC, respectively, *p* < 0.05]. When further interaction analysis was conducted, we identified interaction effects between left-behind status and bullying victimization on friend satisfaction (*p*_interaction_ = 0.048). Similar interaction effect between bullying victimization and left-behind status on school satisfaction was also found (*p*_interaction_ = 0.004).

**Conclusions:** Bullying victimization was associated with low life satisfaction (including lower family, friends, school, self, and environment satisfaction). There were significant interactions between left-behind status and bullying victimization on friend satisfaction, as well as school satisfaction. Left-behind status of children may exaggerate the impact of bullying victimization on friends/school satisfaction rating.

## Introduction

School bullying, including physical forms (e.g., kicking, hitting), verbal forms (e.g., teasing, called mean names, threatened), or psychological aggression (e.g., isolation or rumors), is a worldwide public health issue (1–4). According to a recent national representative study in China, the prevalence of school bullying victimization among primary school students (Grade 1–6) is the highest when compared with middle school (Grade 7–9) or high school students (Grade 11–12) (5). A further study in China showed that compared with high grade children (Grade 10), Grade 6 students have a 5.03-fold risk of being bullied (6). These findings indicate that bullying is likely to occur and is a significant concern for primary school students. Mounting evidence has demonstrated that bullying victimization increases the risk of negative and detrimental mental health problems, such as psychological distress, low life satisfaction, depression, and suicidality (7–10). These negative impacts are detrimental and progress into adulthood (11, 12).

Life satisfaction is a subjective assessment of life quality and is usually achieved by getting individual general needs, goals, or desires satisfied (13). If these basic but important needs or goals are not met or achieved, this usually results in unhappiness or contributes to further mental problems (14, 15). For school age students, their life satisfactions include different domains of life, namely, family, friends, school, self, and environment (16). Several studies have explored the association between bullying victimization and life satisfaction, demonstrating that bullying is associated with significantly lower life satisfaction (10, 17–19).

The left-behind children (LBC) phenomenon refers to the status of a child or adolescent with one or both their parents leaving their home or hometown for work for at least 6 months (20–23). The LBC phenomenon is highly prevalent in major labor export provinces, especially in central and western provinces (24). Hunan Province is a relatively deprived socioeconomic province, and the LBC phenomenon is quite common in Hunan Province. It has been estimated that there were 4.3511 million LBC in the rural area of Hunan Province according to a national survey conducted in 2010 (24). Also, a recent systematic review showed that 91 of 111 studies investigating health impacts of left-behind status were conducted in China (20). LBC are vulnerable to many health problems, such as depressive symptoms (25), oral health problems (26, 27), and delayed growth (28). Notably, emotional, behavioral, and metal health problems in LBC have been comprehensively examined in different countries. Measures have included bullying victimization, life satisfaction, depression, and suicide attempts (11, 20, 29–31). In previous studies, left-behind status or experience was shown to be a moderator of association between children's or teachers' behavior and students' mental traits (32, 33). For example, a school-based health survey in China conducted in 2017 showed that the association between self-esteem and aggressive behavior was more profound in participants with left-behind experiences, and this study found significant interactions between left-behind experience and self-esteem on aggressive behaviors (32). No previous studies have examined the moderating role of left-behind status on the association between bullying victimization and life satisfaction.

In summary, bullying victimization is associated with significantly lower life satisfaction, and the left-behind status of children is likely to be a moderator in the association between bullying victimization and life satisfaction; in other words, there could be interaction effects between left-behind status and bullying victimization on life satisfaction and its different domains. Hunan Province is one of the provinces with the highest number of LBC in China. Bullying victimization and life satisfaction among LBC have been previously examined in Hunan Province. However, no previous studies have explored the interaction between bullying victimization and left-behind status in relation to life satisfaction in children, including family, friends, school, self, and environment satisfaction.

Based on the above discussion, we have proposed two hypotheses as follows: (1) bullying victimization is associated with lower life satisfaction, including the five domains (family, friends, school, self, and environment satisfaction) and (2) there are significant interactive effects between left-behind status and school bullying on life satisfaction (family, friends, school, self, and environment satisfaction).

To examine the hypothesis, we conducted a cross-sectional study on the children of Hunan Province, China, to verify the association of bullying victimization and life satisfaction. We also explored the interactive effects between left-behind status and school bullying on different domains of life satisfaction (family, friends, school, self, and environment satisfaction).

## Materials and Methods

### Location

This present study was conducted in Hunan Province, one of Central China's most populated provinces, with 68.22 million residents and an area of 211,800 km^2^ in 2016. Hunan Province exports large quantities of labor force to other coastal developed cities every year. The left-behind phenomenon is quite common in children, especially in rural or western areas of Hunan Province. According to a national survey in 2010, the number of LBC in rural Hunan Province is estimated to be 4.3511 million (24).

### Participants

A cross-sectional study was conducted among primary school students in three different districts in western Hunan Province in July 2018 using convenience sampling. At first, a total of 820 primary school students and 93 junior high school students were investigated. The sample of the junior high school students was limited, so in the present study, we only involved primary school students. Among these 820 primary school children, 10 children's questionnaires were incomplete (no data of life satisfaction). Therefore, the final sample size in this study was 810. The study was approved by the Research Ethical Review Committee Board of Hunan Normal University (2019-88). Written informed consent was obtained from all participants and their parents or other legal guardians prior to data collection.

Since the official announcement of the “Compulsory Education Law of the People's Republic of China” in April 1986, all children in China are required to receive a 9-year compulsory education in primary and junior middle school (primary school: from Grade 1 to 6, middle school: from Grade 7 to 9). In addition, previous studies in China have found that compared with high school or middle school students, primary school students have a significantly higher risk of being bullied (5, 6). Therefore, we chose primary school students (Grade 1–6) to explore the associations between bullying victimization, left-behind status, and life satisfaction.

### Measurements

#### Bullying Victimization

For bullying victimization, we used the Chinese version of the revised Bully/Victim Questionnaire developed by Dan Olweus (34). We asked the participants six questions to be answered on a 3-point scale (0 = never, 1 = sometimes, 2 = often): in the past 30 days have you (1) been teased in a hurtful way; (2) been blackmailed; (3) been rejected or isolated by peers; (4) been threatened; (5) been hit, kicked, pushed, shoved around, or locked indoors; and (6) been made fun of due to your physical appearance (34). This questionnaire has proven to have good reliability and validity and has been used widely in China in previous studies (29, 35–37). The Cronbach α coefficient was 0.78 in the current study. Children reported at least one question of being bullied with the option of “often” in the past month being defined as being bullied as outlined in previous studies (36, 38).

#### Left-Behind Status

In this study, we used the definition of left-behind as defined in previous studies (20, 21, 23). When one or both parents migrate from where the child lives and leaves behind the child to live alone or with grandparents or other relatives for at least 6 months, then, the child is categorized as LBC (21–23). For further analysis, we divided the subjects into two groups: LBC with one parent migration group and LBC with migration of both parents group.

#### Multidimensional Students' Life Satisfaction Scale

Life satisfaction was assessed using the Multidimensional Students' Life Satisfaction Scale (MSLSS) Chinese version, with a Cronbach α coefficient of 0.90 and a retest reliability of 0.869 obtained in previous studies (39, 40). The MSLSS has five subscales to measure five domains of life satisfaction: family, school, friends, environment, and self-satisfaction. The subscales have acceptable internal consistency and were validated in previous studies for children (39, 40). These are useful and widely used for the assessment of students' life satisfaction (16, 41–43). The Cronbach's alpha value of MSLSS in the present study was 0.869.

### Statistical Analysis

The characteristics of quantitative variables are described as median and interquartile range (IQR) since the variables were not normally distributed and were categorical variables by number and percentages. Chi-square tests were used to compare the categorical variables between boys and girls, and independent-samples Mann–Whitney *U* tests were used to compare the quantitative variable differences between boys and girls. The associations between bullying victimization and life satisfaction (including its domains: family, friends, school, self, environment satisfaction) were examined by multivariate linear regression analyses with adjustments for age, sex, and ethnicity. The association between left-behind status and bullying victimization was examined using univariate and multivariate logistic regression analyses with adjustments for age, sex, ethnicity, education level of the father, and education level of the mother. Stratified analyses of the left-behind status of association between bullying and life satisfaction (including its domains: family, friends, school, self, environment satisfaction) were conducted, and then the interactions were tested using a multivariate general linear model including the interaction term with age, sex, and ethnicity as covariates. In the stratified analyses in our study, the interaction was defined as a different effect size in the subgroups, but the interaction was significant (*p*_interaction_ < 0.05) (44). For the significant interaction effects between left-behind status and bullying victimization, the adjusted means and standard errors of friend and school satisfaction score in different groups were obtained using general linear model with age, ethnicity, and sex adjusted (presented in the figure) (45–47). With regard to missing data in the present study, among these 820 primary school children, 10 children's questionnaires were incomplete (no data of life satisfaction). For the multivariate analysis, missing data were excluded for the statistical analysis (9). All statistical analyses were conducted using IBM SPSS 20.0 for Windows (SPSS Inc., Chicago, IL, USA). *p* < 0.05 was considered statistically significant.

## Results

### General Characteristics

A total of 810 children were involved in the present study; the median (IQR) age was 9 (7, 10) years, the median (IQR) bullying score was 0 (0, 2), and the median (IQR) MSLSS score was 24.03 (21.78, 25.98). There were 454 boys (56.0%) and 356 girls (44%). The prevalence of school bullying was 8.5% (8.8 vs. 8.1% for boys and girls, respectively). For the specific bullying victimization behaviors, no significant differences were observed between the boys and girls (*p* > 0.05). Boys reported significantly higher self-satisfaction than girls (median score was 4.86 vs. 4.57, *p* = 0.042). The prevalence of LBC was 44.3% (44.1 and 44.7% for boys and girls, respectively). Among the LBC, 179 children had only one parent migrated for work, and 180 children had both parents migrated (Table 1).

**Table 1:** The general characteristics of the study population.

**Variables**	**Group**	**Boys**	**Girls**	**Total**	***P***
		**median (IQR)/*N* (%)**	**median (IQR)/*N* (%)**	**median (IQR)/*N* (%)**	
Age		9 (7, 10)	9 (7, 10)	9 (7, 10)	0.471
Ethic groups	Han Ethnicity	399 (87.9%)	297 (83.4%)	696 (85.9%)	0.070
	Others	55 (12.1%)	59 (16.6%)	114 (14.1%)	
Education of father	Junior high school and below	193 (42.5%)	133 (37.4%)	326 (40.2%)	0.163
	High school and above	237 (52.2%)	195 (54.8%)	432 (53.3%)	
	Not reported	24 (5.3%)	28 (7.9%)	52 (6.4%)	
Education of mother	Junior high school and below	227 (50%)	171 (48%)	398 (49.1%)	0.446
	High school and above	198 (43.6%)	154 (43.3%)	352 (43.5%)	
	Not reported	29 (6.4%)	31 (8.7%)	60 (7.4%)	
School bullying	No	414 (91.2%)	327 (91.9%)	741 (91.5%)	0.737
	Yes	40 (8.8%)	29 (8.1%)	69 (8.5%)	
Been teased in a hurtful way	Never	304 (67%)	224 (62.9%)	528 (65.2%)	
	Sometimes	133 (29.3%)	116 (32.6%)	249 (30.7%)	0.231
	Often	17 (3.7%)	16 (4.5%)	33 (4.1%)	
Been blackmailed	Never	424 (93.4%)	325 (91.3%)	749 (92.5%)	
	Sometimes	23 (5.1%)	24 (6.7%)	47 (5.8%)	0.261
	Often	7 (1.5%)	7 (2%)	14 (1.7%)	
Been rejected or isolated by peers	Never	367 (80.8%)	285 (80.1%)	652 (80.5%)	0.781
	Sometimes	74 (16.3%)	58 (16.3%)	132 (16.3%)	
	Often	13 (2.9%)	13 (3.7%)	26 (3.2%)	0.191
Been threatened	Never	389 (85.7%)	293 (82.3%)	682 (84.2%)	
	Sometimes	54 (11.9%)	53 (14.9%)	107 (13.2%)	
	Often	11 (2.4%)	10 (2.8%)	21 (2.6%)	
Been hit, kicked, pushed, shoved around, or locked indoors	Never	381 (83.9%)	297 (83.4%)	678 (83.7%)	0.850
	Sometimes	59 (13%)	50 (14%)	109 (13.5%)	
	Often	14 (3.1%)	9 (2.5%)	23 (2.8%)	
Been made fun of due to my physical appearance	Never	406 (89.4%)	315 (88.5%)	721 (89%)	0.670
	Sometimes	35 (7.7%)	31 (8.7%)	66 (8.1%)	
	Often	13 (2.9%)	10 (2.8%)	23 (2.8%)	
Left-behind status (three categories)	Non-left-behind	254 (55.9%)	197 (55.3%)	451 (55.7%)	0.595
	One parent migration	95 (20.9%)	84 (23.6%)	179 (22.1%)	
	Both parents migration	105 (23.1%)	75 (21.1%)	180 (22.2%)	
Left-behind status (two categories)	Non-left-behind	254 (55.9%)	197 (55.3%)	451 (55.7%)	0.862
	Left-behind	200 (44.1%)	159 (44.7%)	359 (44.3%)	
Bullying score		0 (0, 2)	1 (0, 2)	0 (0, 2)	0.347
Family satisfaction		5 (4.38, 5.63)	5.13 (4.5, 5.63)	5.13 (4.38, 5.63)	0.190
Friend satisfaction		4.56 (4, 5)	4.56 (4.11, 5.11)	4.56 (4.11, 5.00)	0.278
School satisfaction		5.21 (4.43, 5.71)	5.29 (4.57, 5.71)	5.29 (4.57, 5.71)	0.459
Environment satisfaction		4.38 (3.88, 4.88)	4.5 (3.88, 5.00)	4.46 (3.88, 5.00)	0.276
Self-satisfaction		4.86 (4.29, 5.29)	4.57 (4.14, 5.29)	4.71 (4.14,5.29)	**0.042**
Total MSLSS score		23.88 (21.8, 25.96)	24.12 (21.75, 25.99)	24.03 (21.78, 25.98)	0.566

*IQR, interquartile range; MSLSS, multidimensional students' life satisfaction scale. All significant results were marked in bold*.

### Association Between Bullying Victimization and Different Domains of Life Satisfaction

For the associations between bullying victimization and different domains of life satisfaction, the results are outlined in Table 2. With adjustments for potential covariates (age, ethnicity, and sex), bullying victimization was significantly associated with lower family satisfaction (*b* = −0.109, SE = 0.026, *p* < 0.001), friend satisfaction (*b* = −0.091, SE = 0.020, *p* < 0.001), school satisfaction (*b* = −0.095, SE = 0.021, *p* < 0.001), environment satisfaction (*b* = −0.065, SE = 0.022, *p* = 0.004), self-satisfaction (*b* = −0.076, SE = 0.025, *p* = 0.003), and the total MSLSS (b = −0.081, SE = 0.017 *p* < 0.001).

**Table 2:** Association between bullying victimization and different aspects of children's life satisfaction.

**Life satisfaction scores**	**B**	**SE**	***P***
Ln family satisfaction score	−0.109	0.026	<0.001
Ln friend satisfaction score	−0.091	0.020	<0.001
Ln school satisfaction score	−0.095	0.022	<0.001
Ln environment satisfaction score	−0.065	0.022	0.004
Ln self-satisfaction score	−0.076	0.025	0.003
Ln total MSLSS score	−0.081	0.017	<0.001

*Adjustment of age, ethnicity, and sex. Since the scores are not normally distributed, the scores were log transformed for analysis*.

### Interaction Effect of Left-Behind Status and Bullying Victimization on Life Satisfaction

When stratified by left-behind status, we found that the associations between bullying victimization and some life satisfaction domains varied substantially in the LBC and non-LBC groups (Table 3). For the family, environment, and self-satisfaction domains, bullying victimization, no significant interaction effects between left-behind status and bullying victimization on their satisfaction score were noted (*p*_interaction_ > 0.05).

**Table 3:** Interaction effect of left-behind status and bullying victimization on different domains of life satisfaction.

**Different domains of MSLSS**	**Left-behind status**	**School bullying victimization**	**Score**	**b(SE)**	***P***	***p*_**interaction**_**
Family score	Non-LBC	No	5.13 (4.38, 5.75)	−0.119 (0.037)	0.001	0.737
		Yes	4.81 (3.88, 5.38)			
	LBC	No	5.06 (4.50, 5.50)	−0.101 (0.035)	0.004	
		Yes	4.88 (3.88, 5.50)			
Friend score	Non-LBC	No	4.56 (4.11, 5.00)	−0.061 (0.026)	0.021	0.048
		Yes	4.49 (4.00, 4.89)			
	LBC	No	4.56 (4.11, 5.11)	−0.133 (0.03)	<0.001	
		Yes	4.22 (3.33, 4.78)			
School score	Non-LBC	No	5.14 (4.43, 5.71)	−0.041 (0.031)	0.188	0.004
		Yes	5.03 (4.00, 5.71)			
	LBC	No	5.29 (4.71, 5.86)	−0.167 (0.032)	<0.001	
		Yes	4.57 (3.71, 5.29)			
Environment score	Non-LBC	No	4.38 (3.88, 5.00)	−0.051 (0.029)	0.083	0.420
		Yes	4.25 (3.88, 4.75)			
	LBC	No	4.50 (3.88, 5.00)	−0.086 (0.034)	0.013	
		Yes	4.13 (3.63, 4.75)			
Self-score	Non-LBC	No	4.86 (4.29, 5.43)	−0.068 (0.036)	0.060	0.561
		Yes	4.71 (4.00, 5.29)			
	LBC	No	4.71 (4.14, 5.29)	−0.089 (0.035)	0.013	
		Yes	4.29 (3.43, 5.14)			

*Adjustment of age, ethnicity, and sex. Since the scores are not normally distributed, scores were log transformed for analysis. SE, standard error; LBC, left-behind children; MSLSS, multidimensional students' life satisfaction scale*.

**Figure 1:**
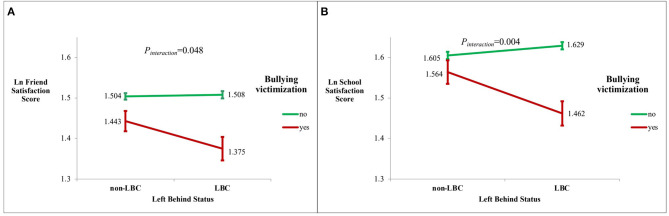
Adjusted means and standard errors of Ln friend and school satisfaction scores stratified by left-behind status and bullying victimization. LBC: left-behind children. Non-LBC: non-left-behind children. Adjusted means and standard errors were estimated under general linear regression model with adjustment for age, ethnicity, and sex. **(A)** Friend satisfaction score and **(B)** school satisfaction score.

For friend satisfaction domain, in the non-LBC group, bullying victimization was negatively associated with the friend satisfaction score; compared with the no bullying group, those with bullying victimization had 0.061 lower score in the Ln value of friend satisfaction score than in non-LBC (*b* = −0.061, SE = 0.026, *p* = 0.021). While in the LBC group, bullying victimization was associated with friend satisfaction score with a more profound effect size of −0.133 (SE = 0.03, *p* < 0.001); this means that the bullying victimization group had 0.133 lower score in the Ln value of school satisfaction score in LBC. The adjusted mean of Ln friend satisfaction score with standard error by left-behind status and bullying victimization is shown in Figure 1A. With further interaction analysis, we found that there was a significant interaction between left-behind status and bullying victimization on friend satisfaction (*p*_interaction_ = 0.048).

For the school domain, a similar significant interaction effect between left-behind status and bullying victimization on school satisfaction was found (*p*_interaction_ = 0.004). In the non-LBC group, bullying victimization was not associated with significant school satisfaction (*b* = −0.041, SE = 0.031, *p* = 0.188). While in the LBC group, bullying victimization was associated with a significant lower level of school satisfaction with an effect size of −0.167 (SE = 0.032, *p* < 0.001), which means that the bullying victimization group had 0.167 lower score in the Ln value of school satisfaction score in LBC. The adjusted mean of Ln school satisfaction score with standard error by left-behind status and bullying victimization is shown in Figure 1B.

## Discussion

Our study found that bullying victimization was negatively associated with life satisfaction (including family, friends, school, environment, and self-satisfaction); in addition, we found that there were interaction effects between left-behind status and school bullying on friend satisfaction, as well as school satisfaction. Left-behind status in children may exaggerate the impact of being bullied associated with different life satisfaction aspects. To the best of our knowledge, this is the first study to explore the interaction effects of bully victimization and left-behind status on life satisfaction. Findings from our study could help develop better strategies for psychological interventions among children suffering bullying victimization, especially for those who are LBC.

Consistent with previous correlational studies (10, 48–52), we verified the negative impact of bullying victimization on different domains or the total score of life satisfaction. The study by Moore et al. on USA adolescents reported pervasive relationships between experiences of bullying and life satisfaction across a variety of domains of satisfaction (48). Also, a Spanish study showed that bullying victimization was associated with specific domains of life satisfaction (including school, family, friends, and self-satisfaction; environment satisfaction was not examined) (49). The studies by Blood and colleagues (52) and Rezapour et al. (51) both reported negative association between bullying victim and total life satisfaction. Slightly different with our finding, Bilic et al. found that children exposed to classic or cyber bullying showed less satisfaction with family and friends, but not significantly associated with school satisfaction in rural and urban areas of Croatia (19); this discrepancy may be due to the different ethnicities or different sample sizes (*n* = 562 and 810 for the study by Bilic and our study, respectively). The underlying reasons of association between bullying victimization and life satisfaction are not clear now, but there are some possible speculations. Firstly, because being bullied may lead to many negative mental problems, such as depression, social anxiety, and even suicide attempts (6, 53–55), these outcomes of being bullied substantially decrease the level of perceived life satisfaction. Secondly, low self-esteem could be a possible mediator for the association between school life satisfaction and being bullied (4). Additionally, the detrimental impact of experience of being bullied in primary school on psychosocial health (including life satisfaction) lasts into adulthood (12). So, it is important to make efforts to prevent bullying victimization and improve individual life satisfaction.

Notably, our study also identified significant interaction effects between left-behind status and bullying victimization on friend satisfaction, as well as school satisfaction. These findings suggest that left-behind status may exaggerate the negative impact of being bullied on friends or school satisfaction. The relationship with peers or friends in childhood is very important; it is reasonable and logical to expect that bullying victimization would affect friend satisfaction (19). Considering the absence of parents' support for LBC, experience of bullying victimization might lead to distrust, sense of insecurity or vulnerability, and subsequently result in lower friends or school satisfaction. So, if the children are suffering from bullying victimization, especially for LBC, efforts for providing enough peer and teacher support could be beneficial for improving their satisfaction or hope in life (56, 57). In addition, the direction of the associations between bullying victimization and life satisfaction requires further investigation. A recent study in China suggested that bullying victimization of LBC could be affected by school life satisfaction (4); so among LBC, the association between bullying victimization and life satisfaction could be bidirectional. Therefore, future in-depth qualitative interviews or intervention studies should be warranted to improve our understanding of this relationship. Although no previous studies have investigated the interaction between bullying victimization and left-behind status on life satisfaction, the study by Barzilay showed that there were significant interaction effects between bullying victimization and parental support and the risk of suicide attempts (54). Left-behind status or parent–child separation is related to a lower level of parent support (58). Therefore, the interaction between left-behind status and bullying victimization on friends/school satisfaction might be due to the low parent support in the LBC. However, the underlying mechanisms need further scientific investigation to confirm this interaction effect. Effective measures and strategies from different stakeholders (including the individual, peers, family, and school) should be implemented to improve these domains of life satisfaction in this specific vulnerable population.

There are some limitations in our study. Firstly, the present study is a cross-sectional study that could only provide associations but not causal inference. Future studies should be longitudinal or even interventions that should be conducted to verify and better understand the associations outlined here. Secondly, this study was only conducted in three districts of Hunan Province by convenience sampling, which may limit generalizations to other areas of China. Thirdly, the current study only involved primary school students; therefore, the results obtained could not be generalizable to children of other ages or migrant status.

## Conclusions

This study indicates that bullying victimization was associated with lower levels of life satisfaction across all domains of life (family, friends, school, self, and environment satisfaction), and left-behind status could interact with bullying victimization to have a negative impact on different domains of life satisfaction (including friends and school satisfaction). It appears that left-behind status in children may exaggerate the impact of bullying victimization on friend satisfaction, as well as school satisfaction.

## Data Availability Statement

The raw data supporting the conclusions of this article will be made available by the authors, without undue reservation.

## Ethics Statement

The studies involving human participants were reviewed and approved by Review Board of Hunan Normal University. Written informed consent to participate in this study was provided by the participants' legal guardian/next of kin.

## Author Contributions

YY, ZZ, and CZ conceived and designed the study. YY, CZ, MX, SY, MZ, SH, and YZe contributed for literature research, data collection, data analysis, manuscript preparation, and manuscript revision. YZh, XY, YW, JSB, and ZZ contributed for literature search and manuscript revision. All authors listed have made a substantial, direct and intellectual contribution to the work, and approved it for publication.

## Conflict of Interest

The authors declare that the research was conducted in the absence of any commercial or financial relationships that could be construed as a potential conflict of interest.

## Publisher's Note

All claims expressed in this article are solely those of the authors and do not necessarily represent those of their affiliated organizations, or those of the publisher, the editors and the reviewers. Any product that may be evaluated in this article, or claim that may be made by its manufacturer, is not guaranteed or endorsed by the publisher.
